# Draft Genome Sequence of Vibrio harveyi Strain GAN1807, Isolated from Diseased Greater Amberjack (Seriola dumerili) Farmed in Nomi Bay, Japan, in 2018

**DOI:** 10.1128/MRA.00629-19

**Published:** 2019-08-22

**Authors:** Yusuke Kato, Takayuki Wada, Hazuki Yamashita, Takuji Ikeda, Kei Nishiyama, Masayuki Imajoh

**Affiliations:** a Graduate School of Integrated Arts and Science, Kochi University, Nankoku, Kochi, Japan; b Department of International Health, Institute of Tropical Medicine, Nagasaki University, Nagasaki, Japan; c School of Tropical Medicine and Global Health, Nagasaki University, Nagasaki, Japan; d Laboratory of Fish Disease, Aquaculture Course, Department of Marine Resource Science, Faculty of Agriculture and Marine Science, Kochi University, Nankoku, Kochi, Japan; e Nomi Fisheries Cooperative Association, Susaki, Kochi, Japan; University of Maryland School of Medicine

## Abstract

Vibrio harveyi is a Gram-negative, bioluminescent bacterium within the family Vibrionaceae. Here, we report the draft genome sequence of V. harveyi strain GAN1807, which was isolated from a diseased greater amberjack (Seriola dumerili) with symptoms of V. harveyi-associated vibriosis in Nomi Bay in Japan.

## ANNOUNCEMENT

Nomi Bay is an enclosed bay located in the south of Shikoku Island, Japan. Extensive mariculture of red sea bream (Pagrus major) and greater amberjack (Seriola dumerili) is carried out in this bay (1). The greater amberjack is branded as “kiwami kanpachi” and widely distributed. A diseased greater amberjack with symptoms of Vibrio harveyi-associated vibriosis was collected on 11 July 2018. A *Vibrio* isolate was grown from the eye lesions of the fish on thiosulfate-citrate-bile salts-sucrose (TCBS) agar and genetically confirmed as V. harveyi, according to our previous study (2). Here, we report the draft genome sequence of this isolate, namely, strain GAN1807.

A single colony of strain GAN1807 grown on TCBS agar was picked for genomic DNA extraction using Genomic-tip 500/G columns and the recommended buffer system (Qiagen) according to the manufacturer’s instructions. Library construction was performed according to the Illumina TruSeq nano DNA library prep kit protocol and sequenced on the Illumina MiSeq platform using the MiSeq reagent kit v3 (600 cycle) with 300-bp paired-end reads. A total of 4,487,036 raw reads were trimmed to remove low-quality reads (quality limit, 0.05; minimum read length, 50 bp), and the remaining 4,169,935 high-quality reads (average length, 228 bp) were assembled using CLC Genomics Workbench 9.5.3 (Qiagen) with default parameters. Annotation of the assembled data was performed using the DDBJ Fast Annotation and Submission Tool (DFAST) pipeline (3). The draft genome of strain GAN1807 was 6,181,216 bp long, with a G+C content of 44.8% and an *N*_50_ value of 176,013 bp, 5,547 predicted protein-coding sequences, 101 tRNA genes, and 4 rRNA operons.

**FIG 1:**
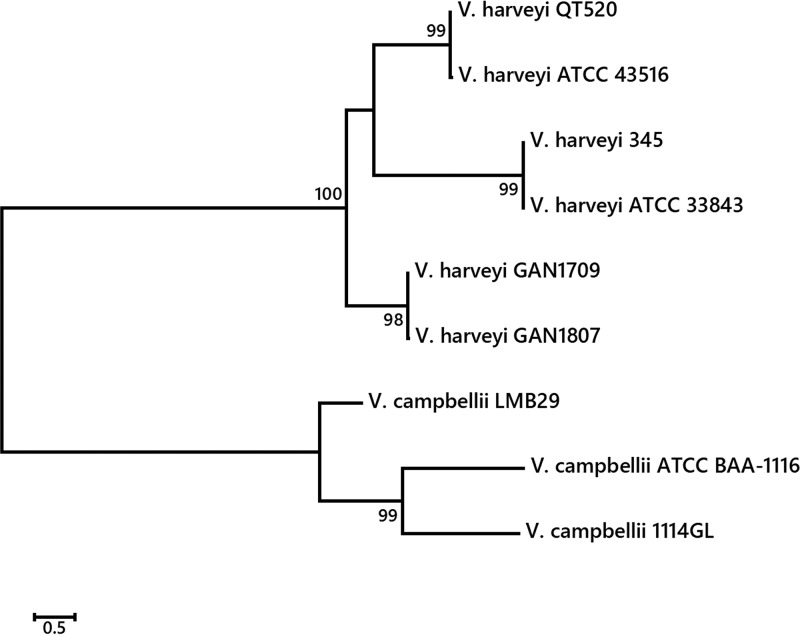
Neighbor-joining phylogenetic tree of six V. harveyi strains and three *V*. *campbellii* strains, based on the concatenated sequences of four housekeeping genes (*toxR*, *vhhA*, *ompK*, and *hsp60*). Bootstrap values are based on 1,000 replicates.

The average nucleotide identity (ANI) between the draft genome of strain GAN1807 and the complete chromosome genomes of the other V. harveyi strains, namely, 345 (GenBank accession numbers CP025537 and CP025538), ATCC 33843 (392 [MAV]) (4) (GenBank accession numbers CP009467 and CP009468), ATCC 43516 (GenBank accession numbers CP014038 and CP014039), and QT520 (5) (GenBank accession numbers CP018680 and CP018681), was calculated using the ANI calculator (6). The ANI values were 98.63% to 98.71%, indicating that strain GAN1807 is closely related to the other four strains, providing that they belong to the same species. A phylogenetic tree was constructed based on the concatenated sequences of four housekeeping genes (*toxR*, *vhhA*, *ompK*, and *hsp60*) of the four V. harveyi strains and three Vibrio campbellii strains, namely, 1114GL (GenBank accession numbers CP019634 and CP019635), ATCC BAA-1116 (GenBank accession numbers CP006605 and CP006606), and LMB29 (GenBank accession numbers CP019293 and CP019294), using the neighbor-joining method in the MEGA6 program (7), to determine their phylogenetic relationships. As shown in Fig. 1, two isolates from a greater amberjack in Nomi Bay, namely, strain GAN1807 from this study and strain GAN1709 from our previous study (2), belonged to the same clade.

### Data availability.

This whole-genome shotgun project has been deposited in DDBJ/GenBank under the accession number BJKR00000000. Raw sequencing reads have been deposited in DDBJ/Sequence Read Archive under the accession number DRR182480.
